# Replication of GWAS Loci Revealed an Increased Risk of *BET1L* and *H19* Polymorphisms with Intracranial Aneurysm

**DOI:** 10.1155/2019/9490639

**Published:** 2019-06-02

**Authors:** Yi Chen, Xiutian Sima

**Affiliations:** ^1^Department of Gastrointestinal Surgery, State Key Laboratory of Biotherapy, West China Hospital of Sichuan University, Chengdu, Sichuan 610041, China; ^2^Department of Neurosurgery, West China Hospital of Sichuan University, Chengdu, Sichuan 610041, China

## Abstract

A genome-wide association study (GWAS) identified that *BET1L* rs2280543 at chromosome 11p15.5 was a susceptibility loci of intracranial aneurysm (IA). Long noncoding RNA *H19*, located in this region, was reported to play a crucial role in the formation of IA. In this study, we aimed to examine whether *BET1L* rs2280543 and potentially functional polymorphisms in *H19* influence the risk of IA. A hospital-based case-control study was performed involving 542 IA patients and 588 age- and gender-matched controls. The *BET1L* rs2280543 and *H19* polymorphisms were genotyped using the TaqMan assay. The *BET1L* rs2280543 CT, CT/TT genotypes, and T allele were associated with an increased risk of IA (CT vs. CC, adjusted OR = 1.43, 95% CI: 1.08-1.90, *P* = 0.01; CT/TT vs. CC, adjusted OR = 1.48, 95% CI: 1.12-1.94, *P* = 0.005; and T vs. C, adjusted OR = 1.44, 95% CI: 1.13-1.83, *P* = 0.003). Similarly, the *H19* rs217727 TT genotype and T allele were associated with an increased risk of IA (TT vs. CC, adjusted OR = 1.90, 95% CI: 1.35-2.67, *P* < 0.001; T vs. C, adjusted OR = 1.38, 95% CI: 1.16-1.64, *P* < 0.001). Combined analyses revealed that the rs2280543 CC-rs217727 CT/TT, rs2280543 CT/TT-rs2735971 GG, and rs217727 CT/TT-rs2735971 GG genotypes were related to the risk of IA. Interaction analysis showed that the 3-loci model of rs2280543-rs217727-rs2839698 contributed to an increased risk of IA. These findings suggest that the GWAS-discovered risk loci *BET1L* rs2280543 may increase IA susceptibility by interacting with lncRNA *H19*.

## 1. Introduction

Intracranial aneurysm (IA) is a balloon-like dilation of the cerebral artery or vein, with an incidence rate of 2-3% in the general population [1]. Rupture of IA can cause 85% of subarachnoid hemorrhage, a death of approximately 50% of the cases, and a severe disability in 30% of the cases [2–4]. It is well accepted that lifestyle diseases including hypertension, cigarette smoking, excessive alcohol intake, and obesity are major risk factors for the development of IA [5–7]. Besides these factors, genetic factors are known to contribute to the physiopathology of IA [8, 9]. Approximately 20% of IA patients have a family history, and a 7-fold higher risk of IA was observed among first-degree relatives compared to second-degree relatives [8, 10–12]. Our previous work also identified some susceptibility loci for the development of IA, such as rs13293512 in the promoter of let-7 and rs4705342 in the promoter of miR-143/145 cluster [13, 14].

Previously, a genome-wide association study (GWAS) identified that 11p15.5 was a risk loci of IA, and a strong association of Bet1 Golgi vesicular membrane trafficking protein like (*BET1L*) rs2280543 at chromosome 11p15.5 with IA susceptibility was observed in a Japanese population [15]. However, little is known to date how the polymorphism influences IA development. Long noncoding RNAs (lncRNAs), single-stranded noncoding RNAs of more than 200 nucleotides in length, were recently found to be implicated in many human diseases, including IA by regulating gene expression at the transcription level or posttranscription level [16–18]. Amounts of lncRNAs were reported to be aberrantly expressed in IA, including *H19* [19, 20]. The lncRNA *H19*, imprinted maternally expressed transcript, is located at chromosome 11p15.5 that is the susceptibility loci of IA [15, 21]. Increasing evidence has shown that *H19* is involved in the process of hypertension and atherosclerosis pathology [22–26], which are important risk factors for the development of IA [5–7].

Genetic variants in *H19* have been demonstrated to play a key role in ischemic stroke [27] and coronary artery disease [28]. TT genotype of rs217727 in *H19* was significantly associated with an increased risk of ischemic stroke, and the association remained after adjusting for confounding risk factors of stroke [27]. The increased risk of the rs217727 TT genotype in *H19* was also observed in coronary artery disease [28]. However, no study to date has investigated the association between single-nucleotide polymorphisms (SNPs) in *H19* and the risk of IA. In this work, a replication study of GWAS-discovered IA risk loci rs2280543 in *BET1L* was performed in a Chinese Han population. Moreover, the effect of potentially functional SNPs in *H19* and their interaction with *BET1L* rs2280543 were also examined.

## 2. Materials and Methods

### 2.1. Ethics Statement

The case-control study was approved by the Institutional Review Board of the West China Hospital of Sichuan University, and written informed consent was provided by each participant. In cases with unconscious or illiterate patients, the written consent was completed by their relatives.

### 2.2. Study Population

The study subjects were enrolled from the West China Hospital of Sichuan University between January 2008 and July 2016, which included 542 IA patients and 588 controls. Detailed information of the study subjects was described previously [13, 14]. For the cases, peripheral blood samples were taken from patients who were definitely diagnosed with IA by digital subtraction angiography. Clinical information was also collected, including age, gender, systolic pressure, diastolic pressure, total cholesterol, triglyceride, high-density lipoprotein cholesterol, low-density lipoprotein cholesterol, the number of aneurysm, ruptured or unruptured aneurysm, and familial aneurysm or not. Patients who had hypertension, head trauma, intracranial atherosclerosis, and/or other nervous system diseases were excluded from the case group. Controls who came to the same hospital for a routine examination of physical condition at the same time period were selected. Controls with previous hypertension and/or some common nervous system diseases, such as stroke, traumatic brain injury, Alzheimer's disease, and Parkinson's disease, were excluded. All subjects were genetically unrelated Han Chinese living in Sichuan province or the surrounding area of southwest China.

### 2.3. SNP Selection

GWAS-identified risk loci *BET1L* rs2280543 was selected. Moreover, potentially functional SNPs in lncRNA *H19* were selected if they met the following criteria: (a) minor allele frequency > 0.1 in the Chinese Han Beijing population of the 1000 Genomes Project and (b) in silico prediction of functional SNPs. Finally, 3 out of 12 SNPs in *H19* were examined in this study since they have potential function, that is, the *H19* rs217727 C allele influencing circulating concentrations of insulin-like growth factor 2 (IGF2) [29]; the rs2839698 CT and TT genotypes resulting in higher levels of H19 [30]; and the rs2735971 A/G having different binding affinity to transcriptional factor CCAAT/enhancer-binding protein alpha (C/EBP*α*).

### 2.4. Genotyping

Genomic DNA was extracted from peripheral blood leukocytes using the Bioteke DNA isolation kit according to the manufacturer's instructions (Bioteke, Beijing, China). Genotyping analysis for the 4 SNPs was performed using the TaqMan allelic discrimination assay on an ABI 7900HT real-time PCR system (Applied Biosystems, CA, USA) [31, 32]. For quality control, the results were read by 2 research staff members blindly. Moreover, about 5% of the samples were selected for Sanger sequencing, and the concordance rates between duplicate samples were 100%.

### 2.5. Statistical Analysis

The SPSS software (version 19.0, SPSS Inc., Chicago, IL) was used for statistical analysis. Hardy-Weinberg equilibrium (HWE) was tested to compare the observed genotype distributions with the expected among controls using the *χ*^2^ test with one degree of freedom. The genotype frequencies of the 4 SNPs between IA cases and controls were compared using the *χ*^2^ test. Associations between the 4 SNPs and IA risk were evaluated by calculating odds ratios (ORs) and their 95% confidence intervals (CIs). ORs were adjusted by age, gender, and familial history of IA using the logistic regression analysis. In multiple comparisons, Bonferroni corrections were used to correct the alpha level of 0.0125 (0.05/4). Multifactor dimensionality reduction (MDR) analysis was carried out to assess *BET1L*-*H19* interaction. All statistical tests were two-sided, and a *P* value < 0.05 was considered to be statistically significant.

## 3. Results

### 3.1. Characteristics of the Study Population

The characteristics of the study population are summarized in Table 1. The mean age of the cases was 51.8 ± 11.8 years, and 39.3% of the patients were male. The mean age of the controls was 51.1 ± 9.1 years, and 36.9% of the controls were male. There was no significant difference between cases and controls according to age (*P* = 0.26) and gender (*P* = 0.41), indicating that the controls were frequency-matched to cases based on age and gender. Among the patients, 86.3% were diagnosed with single intracranial aneurysm, 88.0% with ruptured aneurysm, and 90.0% with nonfamilial aneurysm.

### 3.2. Association of the *BET1L* and *H19* Polymorphisms with IA Risk

The association between the *BET1L* and *H19* polymorphisms and IA risk was presented in Table 2. The genotype frequencies of the 4 SNPs in controls were in HWE (*P* > 0.05). For the *BET1L* rs2280543, an increased risk was significantly associated with the CT genotype (adjusted OR = 1.43, 95% CI: 1.08-1.90, *P* = 0.01) and the CT/TT genotypes (adjusted OR = 1.48, 95% CI: 1.12-1.94, *P* = 0.005) compared with the CC genotype. The similarly increased risk was also observed in the additive model (adjusted OR = 1.40, 95% CI: 1.11-1.78, *P* = 0.005) and allele comparison (adjusted OR = 1.44, 95% CI: 1.13-1.83, *P* = 0.003). For the *H19* rs217727, an increased risk was significantly associated with the TT genotype (adjusted OR = 1.90, 95% CI: 1.35-2.67, *P* < 0.001), T allele (adjusted OR = 1.38, 95% CI: 1.16-1.64, *P* < 0.001), and additive model (adjusted OR = 1.33, 95% CI: 1.13-1.56, *P* < 0.001). For the *H19* rs2839698 and rs2735971, no significant difference of the genotype distributions was found between cases and controls. After stratification analyses according to the number of aneurysms and ruptured-versus-unruptured IA, we failed to find any association between the 4 SNPs and the clinical information (Tables 3 and 4).

### 3.3. Combined Analyses

Combined analyses revealed that individuals with the rs2280543 CC-rs217727 CT/TT genotypes had a 2.35-fold increased risk of IA compared to those with the rs2280543 CC-rs217727 CC genotypes (95% CI: 1.77-3.12, *P* < 0.001); individuals with the rs2280543 CT/TT-rs2735971 GG genotypes had a 1.63-fold increased risk of IA compared to those with the rs2280543 CC-rs2735971 GG genotypes (95% CI: 1.18-2.25, *P* = 0.003); and individuals with the rs217727 CT/TT-rs2735971 GG genotypes had a 1.46-fold increased risk of IA compared to those with the rs217727 CC-rs2735971 GG genotypes (95% CI: 1.09-1.95, *P* = 0.01) (Table 5).

### 3.4. Interaction Analysis

*BET1L*-*H19* interaction analysis showed that the best candidate model was rs2280543-rs217727-rs2839698, with an accuracy of 0.65 and cross-validation consistency of 10/10 (OR = 3.36, 95% CI: 2.60-4.34, *P* < 0.001) (Table 6).

## 4. Discussion

In the present study, we examined whether *BET1L* and *H19* polymorphisms were associated with the risk of IA. We found a statistically significant higher risk of *BET1L* rs2280543 CT, CT/TT genotypes, and T allele among IA patients. The increased risk of IA was also observed in individuals carrying the *H19* rs217727 TT genotype and T allele. Combined analyses revealed that the rs2280543 CC-rs217727 CT/TT, rs2280543 CT/TT-rs2735971 GG, and rs217727 CT/TT-rs2735971 GG genotypes were related to the risk of IA. Interaction analysis showed that the 3-loci model of rs2280543-rs217727-rs2839698 contributed to an increased risk of IA. Using the Quanto software, we computed the statistical power. Our study has more than 81% power if the OR was set at 1.7 under a dominant model. These findings suggest that the GWAS-discovered risk loci *BET1L* rs2280543 may increase IA susceptibility by interacting with lncRNA *H19*.

In 2012, Low et al. conducted a GWAS including 1383 IA patients and 5484 control individuals and identified a susceptibility loci *BET1L* rs2280543 at chromosome 11p15.5 in a Japanese population [15]. Further replication analysis using an additional set of 1048 IA cases and 7212 controls validated this result [15]. In this study, we selected 542 IA patients and 588 age- and gender-matched controls and found that the *BET1L* rs2280543 T variant increased the risk of IA in a Chinese population, which confirmed previous finding [15]. BET1L, encoded by *BET1L* gene, can facilitate the Golgi vesicular membrane trafficking process [33]. However, little is known to date of the biological function of BET1L in IA even though there is a previous report of rs2280543 influencing *BET1L* expression levels in multiple human tissues [34].

The lncRNA *H19*, located on GWAS-discovered IA susceptibility loci of chromosome 11p15.5, was reported to be differentially expressed in IA [19, 20]. Silencing of *H19* can inhibit proliferation and induce apoptosis of vascular smooth muscle cells by regulating the miR-148b/WNT/*β*-catenin pathway [26], whereas overexpression of *H19* can promote proliferation and suppress apoptosis by regulating MAPK and NF-*κ*B signaling pathway [23], implicating the important values of *H19* in atherosclerosis and hypertension that are risk factors for the formation of IA [5–7]. With regard to the *H19* polymorphisms in vascular diseases, Gao et al. reported that the *H19* rs217727 T variant was associated with an increased risk of coronary artery disease in an additive model, dominant model, and recessive model, while the rs2067051 A variant was associated with a decreased risk of coronary artery disease in an additive model and recessive model [28]. Zhu et al. reported that the *H19* rs217727 TT genotype was associated with an increased risk of ischemic stroke, especially in small vessel ischemic stroke [27]. Based on this background, we hypothesized that the *BET1L* rs2280543 may be involved in the occurrence of IA by interacting with lncRNA *H19* polymorphisms. Our findings confirmed this hypothesis. We found that not only the *H19* rs217727 TT genotype but also the combined genotypes of rs2280543 CC-rs217727 CT/TT and rs2280543 CT/TT-rs2735971 GG were associated with an increased risk of IA. Of note, we found that *BET1L*-*H19* interaction (rs2280543-rs217727-rs2839698) conferred the risk of IA. These findings indicate that the *BET1L* rs2280543 increased IA risk partly by interacting with the lncRNA H19 polymorphisms.

We have to admit some limitations in this work. All individuals included in this study were Han Chinese, and thus, these findings cannot be directly extended to other ethnicities until confirmation results were obtained. Gene-gene and gene-environment interactions were important for an association study. *BET1L-H19* interaction was performed in this study. However, gene-environment interaction cannot be assessed due to insufficient data. Control subjects were hospital-based and did not undergo magnetic resonance angiography screening. The possibility of selection bias therefore cannot be ruled out. Additionally, clinical information is incomplete, such as unavailable data of body mass index. Despite these limitations, replication analysis in this study verified the risk factor of *BET1L* rs2280543 in IA occurrence. Moreover, the lncRNA *H19* may singly and interactively with *BET1L* contribute to the susceptibility of IA.

In conclusion, we provided the first evidence that GWAS-discovered IA susceptibility loci *BET1L* rs2280543 may contribute to the risk of IA by interacting with lncRNA *H19* in the Chinese population. Further evaluation of the exact mechanism of *BET1L* and *H19* in the etiology of IA will be of great value, which will be beneficial for us to achieve a comprehensive understanding of GWAS-identified SNPs relevant to the development of IA.

## Figures and Tables

**Table 1:** Demographics of controls and patients with IA.

	Controls *n* = 588	Patients with IA *n* = 542	*P* value
Age (year, mean ± SD)	51.1 ± 9.1	51.8 ± 11.8	0.26
Gender			
Male	217 (36.9)	213 (39.3)	0.41
Female	371 (63.1)	329 (60.7)	
Systolic pressure (mmHg, mean ± SD)	109.1 ± 9.8	107.0 ± 12.9	0.25
Diastolic pressure (mmHg, mean ± SD)	76.5 ± 6.9	76.4 ± 6.2	0.90
TC (mmol/L, mean ± SD)	4.4 ± 0.5	3.9 ± 0.4	<0.001
TG (mmol/L, mean ± SD)	1.2 ± 0.5	1.2 ± 0.3	0.07
HDL-C (mmol/L, mean ± SD)	1.7 ± 0.8	1.4 ± 0.3	<0.001
LDL-C (mmol/L, mean ± SD)	2.1 ± 0.8	2.5 ± 0.7	<0.001
Multiple aneurysm			
Yes		74 (13.7)	
No		468 (86.3)	
Rupture of aneurysm			
Yes		477 (88.0)	
No		65 (12.0)	
Familial aneurysm			
Yes		54 (10.0)	
No		488 (90.0)	

IA: intracranial aneurysm; SD: standard deviation; TC: total cholesterol; TG: triglyceride; HDL-C: high-density lipoprotein cholesterol; LDL-C: low-density lipoprotein cholesterol.

**Table 2:** Association between the *BET1L* and *H19* polymorphisms and IA risk.

Polymorphisms	Controls, *n* = 588 (%)	Patients, *n* = 542 (%)	Adjusted OR (95% CI)^†^	*P* value
*BET1L* rs2280543				
CC	455 (77.4)	380 (70.1)	1.00	
CT	123 (20.9)	147 (27.1)	1.43 (1.08-1.90)	0.01
CT/TT	133 (22.6)	162 (29.9)	1.48 (1.12-1.94)	0.005
Additive model			1.40 (1.11-1.78)	0.005
C allele	1033 (87.8)	907 (83.7)	1.00	
T allele	143 (12.2)	177 (16.3)	1.44 (1.13-1.83)	0.003
*H19* rs217727				
CC	238 (40.5)	182 (33.6)	1.00	
CT	259 (44.0)	230 (42.4)	1.14 (0.87-1.49)	0.36
TT	91 (15.5)	130 (24.0)	1.90 (1.35-2.67)	<0.001
CT/TT	350 (59.5)	360 (66.4)	1.33 (1.03-1.71)	0.03
Additive model			1.33 (1.13-1.56)	<0.001
C allele	735 (62.5)	594 (54.8)	1.00	
T allele	441 (37.5)	490 (45.2)	1.38 (1.16-1.64)	<0.001
*H19* rs2839698				
CC	318 (54.1)	312 (57.6)	1.00	
CT	221 (37.6)	194 (35.8)	0.90 (0.70-1.17)	0.44
TT	49 (8.3)	36 (6.6)	0.80 (0.50-1.27)	0.33
CT/TT	270 (45.9)	230 (42.4)	0.88 (0.69-1.13)	0.32
Additive model			0.88 (0.73-1.06)	0.18
C allele	857 (72.9)	818 (75.5)	1.00	
T allele	319 (27.1)	266 (24.5)	0.89 (0.74-1.08)	0.25
*H19* rs2735971				
GG	413 (70.2)	382 (70.5)	1.00	
AG	153 (26.0)	130 (24.0)	0.86 (0.65-1.14)	0.30
AA	22 (3.7)	30 (5.5)	1.54 (0.87-2.74)	0.14
AG/AA	175 (29.8)	160 (29.5)	0.95 (0.73-1.24)	0.70
Additive model			1.04 (0.85-1.28)	0.68
G allele	979 (83.2)	894 (82.5)	1.00	
A allele	197 (16.8)	190 (17.5)	1.04 (0.83-1.30)	0.74

IA: intracranial aneurysm; OR: odds ratio; CI: confidence interval. ^†^Adjusted by age, gender, and familial history of IA.

**Table 3:** Stratification analyses by the number of aneurysms.

Polymorphisms	Multiple aneurysm, *n* = 74 (%)	Single aneurysm, *n* = 468 (%)	Adjusted OR (95% CI)^†^	*P* value
*BET1L* rs2280543				
CC	59 (79.7)	321 (68.6)	1.00	
CT/TT	15 (20.3)	147 (31.4)	0.56 (0.31-1.02)	0.05
C allele	130 (87.8)	777 (83.0)	1.00	
T allele	18 (12.2)	159 (17.0)	0.68 (0.40-1.14)	0.13
*H19* rs217727				
CC	20 (27.0)	162 (34.6)	1.00	
CT/TT	54 (73.0)	306 (65.4)	1.37 (0.79-2.38)	0.25
C allele	72 (48.6)	522 (55.8)	1.00	
T allele	76 (51.4)	414 (44.2)	1.28 (0.91-1.82)	0.16
*H19* rs2839698				
CC	46 (62.2)	266 (56.8)	1.00	
CT/TT	28 (37.8)	202 (43.2)	0.79 (0.48-1.31)	0.36
C allele	115 (77.7)	703 (75.1)	1.00	
T allele	33 (22.3)	233 (24.9)	0.85 (0.56-1.29)	0.45
*H19* rs2735971				
GG	53 (71.6)	329 (70.3)	1.00	
AG/AA	21 (28.4)	139 (29.7)	0.95 (0.55-1.64)	0.85
G allele	123 (83.1)	771 (82.4)	1.00	
A allele	25 (16.9)	165 (17.6)	0.94 (0.59-1.49)	0.79

IA: intracranial aneurysm; OR: odds ratio; CI: confidence interval. ^†^Adjusted by age, gender, and familial history of IA.

**Table 4:** Stratification analyses by ruptured aneurysm (yes vs. no).

Polymorphisms	Ruptured aneurysm		Adjusted OR (95% CI)^†^	*P* value
	Yes, *n* = 477 (%)	No, *n* = 65 (%)		
*BET1L* rs2280543				
CC	333 (69.8)	47 (72.3)	1.00	
CT/TT	144 (30.2)	18 (27.7)	0.88 (0.49-1.56)	0.65
C allele	797 (83.5)	110 (84.6)	1.00	
T allele	157 (16.5)	20 (15.4)	0.91 (0.55-1.52)	0.72
*H19* rs217727				
CC	160 (33.5)	22 (33.8)	1.00	
CT/TT	317 (66.5)	43 (66.2)	1.05 (0.60-1.82)	0.87
C allele	522 (54.7)	72 (55.4)	1.00	
T allele	432 (45.3)	58 (44.6)	1.01 (0.70-1.47)	0.94
*H19* rs2839698				
CC	270 (56.6)	42 (64.6)	1.00	
CT/TT	207 (43.4)	23 (35.4)	0.71 (0.41-1.21)	0.20
C allele	714 (74.8)	104 (80.0)	1.00	
T allele	240 (25.2)	26 (20.0)	0.74 (0.47-1.16)	0.18
*H19* rs2735971				
GG	335 (70.2)	47 (72.3)	1.00	
AG/AA	142 (29.8)	18 (27.7)	0.92 (0.51-1.64)	0.77
G allele	787 (82.5)	107 (82.3)	1.00	
A allele	167 (17.5)	23 (17.7)	1.04 (0.64-1.68)	0.88

IA: intracranial aneurysm; OR: odds ratio; CI: confidence interval. ^†^Adjusted by age, gender, and familial history of IA.

**Table 5:** Combined analyses of the *BET1L* and *H19* polymorphisms with IA risk.

Combined genotypes	Controls (%)	IA (%)	OR (95% CI)	*P* value
*BET1L* rs2280543-*H19* rs217727				
rs2280543 CC-rs217727 CC	238 (40.5)	121 (22.3)	1.00	
rs2280543 CC-rs217727 CT/TT	217 (36.9)	259 (47.8)	2.35 (1.77-3.12)	<0.001
rs2280543 CT/TT-rs217727 CC	—	61 (11.3)	—	—
rs2280543 CT/TT-rs217727 CT/TT	133 (22.6)	101 (18.6)	1.49 (1.06-2.10)	0.02
*BET1L* rs2280543-*H19* rs2839698				
rs2280543 CC-rs2839698 CC	237 (40.3)	219 (40.4)	1.00	
rs2280543 CC-rs2839698 CT/TT	218 (37.1)	161 (29.7)	0.80 (0.61-1.05)	0.11
rs2280543 CT/TT-rs2839698 CC	81 (13.8)	93 (17.2)	1.24 (0.88-1.76)	0.22
rs2280543 CT/TT-rs2839698 CT/TT	52 (8.8)	69 (12.7)	1.44 (0.96-2.15)	0.08
*BET1L* rs2280543-*H19* rs2735971				
rs2280543 CC-rs2735971 GG	325 (55.3)	265 (48.9)	1.00	
rs2280543 CC-rs2735971 AG/AA	130 (22.1)	115 (21.2)	1.09 (0.81-1.46)	0.59
rs2280543 CT/TT-rs2735971 GG	88 (15.0)	117 (21.6)	1.63 (1.18-2.25)	0.003
rs2280543 CT/TT-rs2735971 AG/AA	45 (7.7)	45 (8.3)	1.23 (0.79-1.91)	0.37
*H19* rs217727-*H19* rs2839698				
rs217727 CC-rs2839698 CC	128 (21.8)	105 (19.4)	1.00	
rs217727 CC-rs2839698 CT/TT	110 (18.7)	77 (14.2)	0.85 (0.58-1.26)	0.42
rs217727 CT/TT-rs2839698 CC	190 (32.3)	207 (38.2)	1.33 (0.96-1.84)	0.09
rs217727 CT/TT-rs2839698 CT/TT	160 (27.2)	153 (28.2)	1.17 (0.83-1.64)	0.38
*H19* rs217727-*H19* rs2735971				
rs217727 CC-rs2735971 GG	175 (29.8)	128 (23.6)	1.00	
rs217727 CC-rs2735971 AG/AA	63 (10.7)	54 (10.0)	1.17 (0.76-1.80)	0.47
rs217727 CT/TT-rs2735971 GG	238 (40.5)	254 (46.9)	1.46 (1.09-1.95)	0.01
rs217727 CT/TT-rs2735971 AG/GG	112 (19.0)	106 (19.6)	1.29 (0.91-1.84)	0.15

IA: intracranial aneurysm; OR: odds ratio; CI: confidence interval.

**Table 6:** Interaction analysis of the *BET1L* and *H19* polymorphisms and risk of IA.

Best candidate models	Accuracy	Cross-validation consistency	OR (95% CI)	*P* value
rs217727	0.55	10/10	1.70 (1.24-2.32)	0.001
rs2280543-rs217727	0.64	10/10	3.10 (2.40-4.01)	<0.001
rs2280543-rs217727-rs2839698	0.65	10/10	3.36 (2.60-4.34)	<0.001

IA: intracranial aneurysm; OR: odds ratio; CI: confidence interval.

## Data Availability

All data included in this study are available upon request by contact with the corresponding author.

## References

[1] Rinkel G. J. E., Djibuti M., Algra A., van Gijn J. (1998). Prevalence and Risk of Rupture of Intracranial Aneurysms. *Stroke*.

[2] Inagawa T. (2005). Trends in surgical and management outcomes in patients with aneurysmal subarachnoid hemorrhage in Izumo City, Japan, between 1980-1989 and 1990-1998. *Cerebrovascular Diseases*.

[3] Ruigrok Y. M., Rinkel G. J. E. (2010). From GWAS to the clinic: risk factors for intracranial aneurysms. *Genome Medicine*.

[4] Suarez J. I., Tarr R. W., Selman W. R. (2006). Aneurysmal subarachnoid hemorrhage. *New England Journal of Medicine*.

[5] Yu-xiang G. U., Chen X.-c., Song D.-l., Leng B., Zhao F. (2006). Risk factors for intracranial aneurysm in a Chinese ethnic population. *Chinese Medical Journal*.

[6] Inagawa T. (2010). Risk factors for the formation and rupture of intracranial saccular aneurysms in Shimane, Japan. *World Neurosurgery*.

[7] Teunissen L. L., Rinkel G. J. E., Algra A., van Gijn J. (1996). Risk factors for subarachnoid hemorrhage: a systematic review. *Stroke*.

[8] Kojima M., Nagasawa S., Lee Y. E., Takeichi Y., Tsuda E., Mabuchi N. (1998). Asymptomatic familial cerebral aneurysms. *Neurosurgery*.

[9] Wang M. C., Rubinstein D., Kindt G. W., Breeze R. E. (2002). Prevalence of intracranial aneurysms in first-degree relatives of patients with aneurysms. *Neurosurgical Focus*.

[10] Bromberg J. E., Rinkel G. J., Algra A. (1995). Subarachnoid haemorrhage in first and second degree relatives of patients with subarachnoid haemorrhage. *BMJ*.

[11] Kim D. H., Van Ginhoven G., Milewicz D. M. (2003). Incidence of Familial Intracranial Aneurysms in 200 Patients: Comparison among Caucasian, African-American, and Hispanic Populations. *Neurosurgery*.

[12] Wills S., Ronkainen A., van der Voet M. (2003). Familial intracranial aneurysms: an analysis of 346 multiplex Finnish families. *Stroke*.

[13] Sima X., Sun H., Zhou P., You C. (2015). A potential polymorphism in the promoter of Let-7 is associated with an increased risk of intracranial aneurysm: a case-control study. *Medicine*.

[14] Sima X., Sun H., Zhou P., You C., Cai B. (2017). Association between functional polymorphisms in the promoter of the miR-143/145 cluster and risk of intracranial aneurysm. *Scientific Reports*.

[15] Low S. K., Takahashi A., Cha P. C. (2012). Genome-wide association study for intracranial aneurysm in the Japanese population identifies three candidate susceptible loci and a functional genetic variant at EDNRA. *Human Molecular Genetics*.

[16] Bertone P., Stolc V., Royce T. E. (2004). Global identification of human transcribed sequences with genome tiling arrays. *Science*.

[17] Mattick J. S. (2005). The functional genomics of noncoding RNA. *Science*.

[18] Qi P., Du X. (2013). The long non-coding RNAs, a new cancer diagnostic and therapeutic gold mine. *Modern Pathology*.

[19] Li H., Wang W., Zhang L. (2017). Identification of a long noncoding RNA-associated competing endogenous RNA network in intracranial aneurysm. *World Neurosurgery*.

[20] Wang W., Li H., Yu L. (2017). Aberrant expression of lncRNAs and mRNAs in patients with intracranial aneurysm. *Oncotarget*.

[21] Brannan C. I., Dees E. C., Ingram R. S., Tilghman S. M. (1990). The product of the H19 gene may function as an RNA. *Molecular and Cellular Biology*.

[22] Kumar S., Williams D., Sur S., Wang J.-Y., Jo H. (2019). Role of flow-sensitive microRNAs and long noncoding RNAs in vascular dysfunction and atherosclerosis. *Vascular Pharmacology*.

[23] Pan J. X. (2017). LncRNA H19 promotes atherosclerosis by regulating MAPK and NF-kB signaling pathway. *European Review for Medical and Pharmacological Sciences*.

[24] Su H., Xu X., Yan C. (2018). LncRNA H19 promotes the proliferation of pulmonary artery smooth muscle cells through AT1R via sponging let-7b in monocrotaline-induced pulmonary arterial hypertension. *Respiratory Research*.

[25] Wang R., Zhou S., Wu P. (2018). Identifying involvement of H19-miR-675-3p-IGF1R and H19-miR-200a-PDCD4 in treating pulmonary hypertension with melatonin. *Molecular Therapy Nucleic Acids*.

[26] Zhang L., Cheng H., Yue Y., Li S., Zhang D., He R. (2018). H19 knockdown suppresses proliferation and induces apoptosis by regulating miR-148b/WNT/*β*-catenin in ox-LDL -stimulated vascular smooth muscle cells. *Journal of Biomedical Science*.

[27] Zhu R., Liu X., He Z. (2018). Long non-coding RNA H19 and MALAT1 gene variants in patients with ischemic stroke in a northern Chinese Han population. *Molecular Brain*.

[28] Gao W., Zhu M., Wang H. (2015). Association of polymorphisms in long non-coding RNA H19 with coronary artery disease risk in a Chinese population. *Mutation Research/Fundamental and Molecular Mechanisms of Mutagenesis*.

[29] Gatford K. L., Heinemann G. K., Thompson S. D. (2014). Circulating IGF1 and IGF2 and SNP genotypes in men and pregnant and non-pregnant women. *Endocrine Connections*.

[30] Yang C., Tang R., Ma X. (2015). Tag SNPs in long non-coding RNA H19 contribute to susceptibility to gastric cancer in the Chinese Han population. *Oncotarget*.

[31] Bondagji N. S., Morad F. A., al-Nefaei A. A. A. (2017). Replication of GWAS loci revealed the moderate effect of TNRC6B locus on susceptibility of Saudi women to develop uterine leiomyomas. *Journal of Obstetrics and Gynaecology Research*.

[32] Li S., Hua Y., Jin J. (2016). Association of genetic variants in lncRNA H19 with risk of colorectal cancer in a Chinese population. *Oncotarget*.

[33] Xu Y., Wong S. H., Zhang T., Subramaniam V. N., Hong W. (1997). GS15, a 15-Kilodalton Golgi SolubleN-Ethylmaleimide-sensitive Factor Attachment Protein Receptor (SNARE) Homologous to rbet1. *Journal of Biological Chemistry*.

[34] Liu B., Wang T., Jiang J. (2018). Association of BET1L and TNRC6B with uterine leiomyoma risk and its relevant clinical features in Han Chinese population. *Scientific Reports*.

